# Grape Seed Proanthocyanidins Exert a Neuroprotective Effect by Regulating Microglial M1/M2 Polarisation in Rats with Spinal Cord Injury

**DOI:** 10.1155/2022/2579003

**Published:** 2022-08-04

**Authors:** Wen-zhao Liu, Zhan-jun Ma, Ji-he Kang, Ai-xin Lin, Zhao-heng Wang, Hai-wei Chen, Xu-dong Guo, Xue-gang He, Xue-wen Kang

**Affiliations:** ^1^The Second Clinical Medical College, Lanzhou University, Lanzhou, Gansu 730030, China; ^2^Department of Orthopedics, Lanzhou University Second Hospital, Lanzhou, Gansu 730030, China; ^3^Louvain Drug Research Institute, Advanced Drug Delivery and Biomaterials, Université Catholique de Louvain, UCLouvain, 1200 Brussels, Belgium; ^4^The International Cooperation Base of Gansu Province for the Pain Research in Spinal Disorders, Gansu 730000, China

## Abstract

Spinal cord injury (SCI) is a highly disabling disorder for which few effective treatments are available. Grape seed proanthocyanidins (GSPs) are polyphenolic compounds with various biological activities. In our preliminary experiment, GSP promoted functional recovery in rats with SCI, but the mechanism remains unclear. Therefore, we explored the protective effects of GSP on SCI and its possible underlying mechanisms. We found that GSP promoted locomotor recovery, reduced neuronal apoptosis, increased neuronal preservation, and regulated microglial polarisation *in vivo*. We also performed *in vitro* studies to verify the effects of GSP on neuronal protection and microglial polarisation and their potential mechanisms. We found that GSP regulated microglial polarisation and inhibited apoptosis in PC12 cells induced by M1-BV2 cells through the Toll-like receptor 4- (TLR4-) mediated nuclear factor kappa B (NF-*κ*B) and phosphatidylinositol 3-kinase/serine threonine kinase (PI3K/AKT) signaling pathways. This suggests that GSP regulates microglial polarisation and prevents neuronal apoptosis, possibly by the TLR4-mediated NF-*κ*B and PI3K/AKT signaling pathways.

## 1. Introduction

Spinal cord injury (SCI) is a serious neurological disorder worldwide, with devastating effects [1]. The pathological processes of SCI can be divided into primary and secondary injury. Primary injury refers to injury caused by external forces acting directly on the spinal cord [2, 3], whereas secondary SCI is progressive and delayed, including inflammation, ischemia, apoptosis, oedema, and local reactive gliosis [4, 5]. As the primary injury is irreversible, current treatments mainly focus on the symptoms of secondary injury, such as serious neuroinflammation [6].

Microglial activation is the key to the pathogenesis of secondary inflammatory injuries [7, 8]. Activated microglia are very important for repairing central nervous system (CNS) injuries [9, 10]. When unstimulated, microglia are of great significance for monitoring and regulating CNS homeostasis, neuronal regeneration, and proliferation [11]. Microglia can differentiate into two different phenotypes (M1/M2) to cope with the interference of various microenvironments [12]. Overactivation of M1 microglia prevents neuronal regeneration and leads to neuronal damage [13]. M1 microglia can release proinflammatory factors that can aggravate injury and hinder cell repair after CNS injury and disorders. Alternatively, M2 microglia release anti-inflammatory factors [14–16]. Therefore, inhibition of M1 microglial activation is an effective therapeutic strategy for SCI. However, researchers have recently found that inhibition of M1 polarisation alone does not provide overall benefits. A more promising approach is to convert overactivated M1 microglia to the M2 phenotype following SCI [17].

Toll-like receptor 4 (TLR4), a transmembrane receptor, is highly expressed in microglia in the CNS [18, 19]. Stimulation with various ligands, including lipopolysaccharide (LPS), can activate downstream molecules. LPS can bind to LPS-binding protein (LBP), which is mainly produced by the liver. The LPS–LBP complex forms a larger complex with cluster of differentiation 14 (CD14) and activates the TLR4/myeloid differentiation factor 2 (MD2) complex, following which the intracellular part of TLR4 can activate its downstream myeloid differentiation primary reactive protein 88 (MyD88) and NF-*κ*B and inhibit the PI3K/AKT pathway via MyD88 recruitment [20]. Activated NF-*κ*B affects the transcription of proinflammatory cytokines, leading to neuroapoptosis and neuroinflammation [21–24]. Therefore, a viable therapeutic strategy for treating SCI involves inhibiting neuroapoptosis and neuroinflammation by inactivating this signaling pathway.

**Figure 1:**
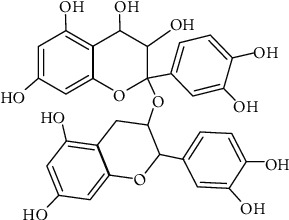
The chemical structure of GSP.

Proanthocyanidins, which are complex flavonoid polymers (Figure 1), are natural polyphenols found in many foods and beverages. The most abundant source is grape (*Vitis vinifera*) seeds, yielding grape seed proanthocyanidins (GSP) [25–28]. Proanthocyanidins have anti-inflammatory, antiapoptotic, antioxidant, and free-radical-scavenging properties [29–31]. In 2017, a study showed that proanthocyanidins had a positive effect on LPS-induced depression-like behavior [32]. In 2022, GSP was reported to have a promising role in modulating bisphenol A-induced neurotoxicity and neuroinflammation [33]. Nevertheless, whether GSP regulates microglial polarisation remains unclear.

Our study suggests that GSP promotes microglial M2 polarisation by targeting TLR4, thus promoting the recovery of locomotor function in rat models of SCI. These findings indicate a promising therapeutic target for SCI treatment.

## 2. Materials and Methods

### 2.1. Materials

GSP with a purity ≥ 95% was obtained from Solarbio (Beijing, China). LPS was obtained from Sigma-Aldrich. High-glucose DMEM and fetal bovine serum (FBS) were obtained from Gibco/BRL (Grand Island, NY, USA). CCK-8, fluorescein isothiocyanate/propidium iodide (FITC/PI), and nitric oxide (NO) assay kits were purchased from Beyotime (Shanghai, China). Antibodies against CD86 and CD206 were obtained from Santa Cruz Biotechnology (Dallas, TX, USA). Antibodies against GFAP, NeuN, I*κ*B*α*, Iba1, TLR4, MyD88, p-I*κ*B*α*, p-NF-*κ*B p65, p-PI3K, PI3K, p-AKT, NF-*κ*B p65, and AKT were purchased from Cell Signalling Technology (Danvers, MA, USA). Antibodies against *β*-actin, ARG1, BCL-2, inducible nitric oxide synthase (iNOS), BAX, IL-10, TNF-*α*, and cleaved caspase-3 were purchased from Proteintech Group Inc. (Chicago, IL, USA).

### 2.2. Animal Model of SCI and GSP Administration

**Figure 2:**
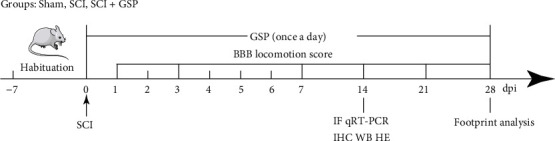
Timeline of the experimental protocol.

Sixty specific pathogen-free adult Sprague-Dawley rats (female, 230 ± 20 g) were obtained from the Animal Experimental Centre of Lanzhou University. All rats were housed in separate cages under controlled housing conditions (23°C ± 2°C, 50% ± 5%humidity, and 12-h light-dark cycle). The animals were randomly divided into three groups (Figure 2): (1) sham, (2) SCI, and (3) SCI+40 mg/kg GSP (*n* = 20 per group). The modified Allen method was adopted to establish the SCI models [34]. Briefly, rats were anesthetized with pentobarbital (1%, 40 mg/kg). An approximately 2.0 cm midline incision was made, and the paraspinal muscles over the area of the vertebral T8–10 level were bluntly dissected. A T9 vertebral laminectomy was subsequently performed, and a spinal cord impactor was used to injure the rats using a 10 g rod falling freely from a height of 10 cm. In the sham rats, the spinal cord was only exposed without causing SCI. The injured rats exhibited involuntary hind limb spasms and apnoea and wriggled tails, indicating that the SCI models were successful. The rats were voided twice a day. In addition, the rats in the GSP-treated group were intraperitoneally injected with GSP (40 mg/kg or 67.34 *μ*M/kg) once a day, and GSP was absorbed through the mesenteric vein. The remaining rats received the same volume of saline. All animal care and husbandry procedures were approved by the Animal Ethics Committee of Lanzhou University Second Hospital and were performed in accordance with the National Institutes of Health Guide for the Care and Use of Laboratory Animals guidelines.

### 2.3. Locomotor Recovery Assessment

The Basso, Beattie, and Bresnahan (BBB) scale was used to evaluate the extent of locomotor recovery experienced by rats following SCI [35], with scores in the range of 0-21. A score below 21 indicates impaired locomotor ability. Footprint analysis was adopted to evaluate motor coordination by immersing the hindlimb and forelimb in red and purple dyes, respectively. The results were obtained as the rats passed through a runway lined with white paper. The aforementioned tests were performed by two trained investigators who were blinded to the treatment regimens.

### 2.4. Terminal Deoxynucleotidyl Transferase dUTP Nick-End Labelling (TUNEL) Assay

Cellular apoptosis levels were determined using a TUNEL assay kit (Beyotime) according to the manufacturer's instructions. The cells were processed as previously described. The number of TUNEL-positive apoptotic cells was determined by calculating the mean of three randomly selected fields.

### 2.5. Annexin V-FITC/PI Assays

The cells were treated and centrifuged at 300 × *g* for 5 min and subsequently washed three times with precooled phosphate-buffered saline (PBS). Following which the cells were centrifuged at 300 × *g* at 4°C for 5 min after each wash. PBS was discarded, and the cells were resuspended in 100 *μ*L of binding buffer. Next, Annexin V-FITC (5 *μ*L) and PI (10 *μ*L) solutions were sequentially added, and cells were stained at room temperature in the dark for 15 min. The cells were detected using flow cytometry.

### 2.6. Cell Viability and Morphological Analysis

BV2 cells were treated with 3.125, 6.25, 12.5, 25, 50, 100, and 200 *μ*M GSP for 24 h. Another batch of cells was treated with LPS (100 ng/mL) for 24 h, followed by treatment with different concentrations of GSP for 24 h. CCK-8 solution (10 *μ*L) was added to each well and maintained for 90 min. Finally, absorbance at 450 nm was measured using a microplate reader (Bio-Rad, Hercules, CA, USA). For morphological analysis, the cells were photographed using an EVOS XL Core Cell Imaging System (Semefeld, Waltham, MA, USA) at a magnification of ×100.

### 2.7. BV2 Cell Culture and Treatment

BV2 cells were cultured in MEM containing 10% FBS (Gibco) and 1% penicillin/streptomycin in a humidified 5% CO_2_ atmosphere. They were passaged with 0.25% trypsin at approximately 80% confluence. BV2 cells were pretreated with 100 ng/mL of LPS for 24 h, followed by treatment with different concentrations of GSP (3.125, 6.25, and 12.5 *μ*M) for 24 h. To explore the underlying mechanism of GSP, the TLR4-specific inhibitor TAK242 (1 *μ*M, 60 min) was added to the BV2 cells before adding GSP.

### 2.8. Microglia/Neuron Coculture

A Transwell coculture system (0.4 *μ*m pores; Corning, USA) incubated in a 24-well plate was employed in this study. PC12 cells were cultured in DMEM. BV2 cells were pretreated with 100 ng/mL LPS for 24 h, followed by treatment with 12.5 *μ*M GSP for 24 h. BV2 cells were then seeded into inserts, placed on the PC12 monolayer at the bottom of the well, and cultured for 24 h.

### 2.9. Western Blotting (WB)

Spinal cord tissues or cultured cells were lysed in RIPA buffer supplemented with phosphatase inhibitors and protease. The lysates were centrifuged at 12,000 rpm (4°C, 30 min), and the supernatants were collected. Protein concentrations were determined using a bicinchoninic acid kit. Proteins were separated by 10% or 12% sodium dodecyl polyacrylamide gel electrophoresis and transferred onto PVDF membranes. After blocking with 5% nonfat milk, the membranes were incubated overnight at 4°C with the following primary antibodies: iNOS (anti-rabbit, 1 : 1250), Arg1 (anti-rabbit, 1 : 1000), TNF-*α* (anti-rabbit, 1 : 1000), IL-10 (anti-rabbit, 1 : 1000), cleaved caspase-3 (anti-rabbit, 1 : 1000), Bax (anti-mouse, 1 : 1000), Bcl-2 (anti-mouse, 1 : 2000), and *β*-actin (anti-mouse, 1 : 1000) from Proteintech; CD86 (anti-mouse, 1 : 1000) and CD206 (anti-mouse, 1 : 1000) from Santa Cruz Biotechnology; and p-NF-*κ* B-p65 (anti-rabbit, 1 : 1000), NF-*κ*B-p65 (anti-rabbit, 1 : 1000), I*κ*B*α* (anti-rabbit, 1 : 1000), p-I*κ*B*α* (anti-rabbit, 1 : 1000), PI3K (anti-rabbit, 1 : 1000), p-PI3K (anti-rabbit, 1 : 1000), AKT (anti-rabbit, 1 : 1000), and p-AKT (anti-rabbit, 1 : 1000) from Cell Signalling Technology. The membranes were incubated with goat anti-rabbit/mouse antibody (1 : 4000; Proteintech) and labeled with horseradish peroxidase for 1.5 h the next day. The protein signals were detected using an imaging system.

### 2.10. NO Assay

The ability of microglia to produce NO was evaluated by measuring the release of nitrite from the culture supernatant. BV2 cells were pretreated with 100 ng/mL LPS for 24 h, followed by treatment with different concentrations of GSP for 24 h. A NO assay kit (Beyotime) was used to detect NO production according to the manufacturer's instructions.

### 2.11. Immunofluorescence Staining (IF)

Spinal cord tissues were removed, embedded in paraffin, and cut into longitudinal 4 *μ*m-thick sections. After being blocked with 10% goat serum, the tissue sections were incubated overnight at 4°C with the following primary antibodies: anti-NeuN (1 : 200), anti-GFAP (1 : 300), anti-Iba1 (1 : 300), anti-CD86 (1 : 200), and anti-CD206 (1 : 200). For cell IF, the cells were placed on slides, fixed in 4% paraformaldehyde, and infiltrated with 0.5% Triton X-100. The cells were blocked with 10% goat serum and then incubated with anti-iNOS (1 : 250), anti-CD206 (1 : 250), anti-p-NF-*κ*B-p65 (1 : 300), and p-AKT (1 : 300) primary antibodies overnight at 4°C. The next day, the tissue sections or cells on the slides were treated with the secondary antibodies for 2 h, stained with DAPI, and photographed under a fluorescence microscope.

### 2.12. Quantitative Real-Time Polymerase Chain Reaction (qRT-PCR)

RNA was extracted from spinal cord tissue using TRIzol Reagent (Qiagen, CA, USA). A reverse transcription kit (Takara, China) was used to synthesize complementary DNA (cDNA) following a standard protocol with the LC96 System (Roche, Pleasanton, CA, USA) and quantified with SYBR Green (Takara). The relative expression levels of the different genes were normalized to GAPDH using the 2^−*ΔΔ*Ct^ approach. Each experiment was performed in triplicate. The sequences of the primers are listed in Table 1.

### 2.13. Hematoxylin and Eosin (HE) Staining

The processed tissue sections were stained with hematoxylin for 70 s, differentiated in 1% hydrochloric acid, and stained with eosin for 100 s. Images were obtained under a light microscope.

### 2.14. Immunohistochemistry (IHC)

After dewaxing, the paraffin tissue sections were subjected to antigen retrieval with sodium citrate buffer, blocked with 10% serum, and incubated with anti-cleaved caspase-3 antibody (1 : 100) overnight at 4°C. The following day, sections were rinsed with PBS three times, followed by treatment with secondary antibody for 1.5 h at 37°C. Diaminobenzidine and hematoxylin were used to visualize the antibody staining. Images were obtained using a light microscope.

### 2.15. Statistical Analysis

All experiments in this study were repeated at least three times. All data are presented as mean ± standard deviation and were analyzed using SPSS software (version 22.0; IBM, Armonk, NY, USA). When comparing two groups, the unmatched Student's *t*-test was used. One-way analysis of variance (ANOVA) and Tukey's multiple comparisons test were used for more than two groups.

## 3. Results

### 3.1. Effect of GSP on the Recovery of Locomotion in SCI Rats

**Figure 3:**
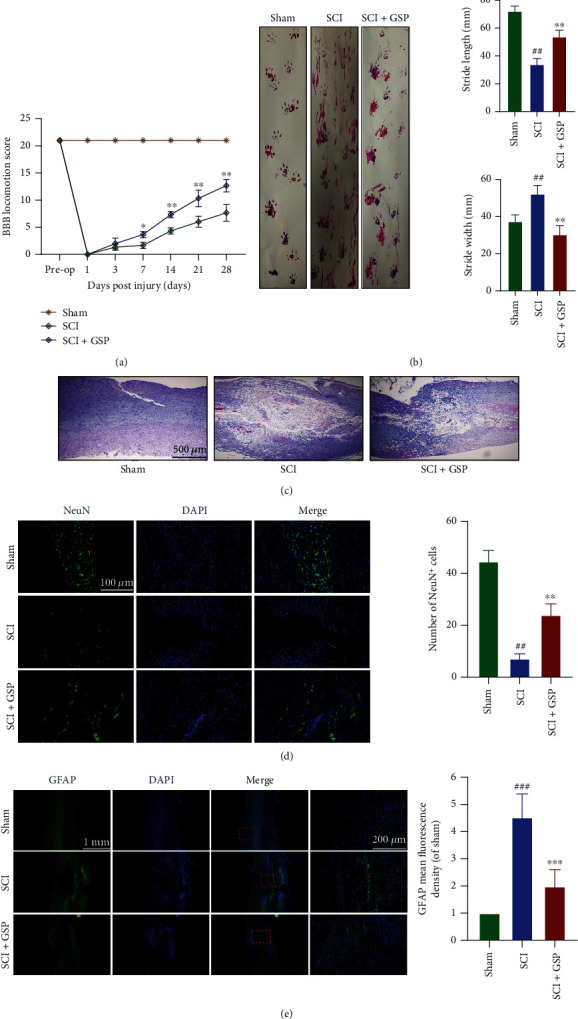
Effect of GSP on locomotor function recovery in SCI rats. (a) BBB score in the three groups at 28 dpi (*n* = 6/group). (b) Footprints and their quantification analysis in each rat at 28 dpi. Purple: frontpaw; Red: hindpaw (*n* = 5/group). (c) Representative HE staining images. (*n* = 3/group). (d) IF and quantitative data for NeuN (green) at 14 dpi (*n* = 3/group). (e) IF and quantitative data for GFAP (green) at 14 dpi. (*n* = 3/group). ^##^*p* < 0.01, ^###^*p* < 0.001 vs. sham group, ^∗∗^*p* < 0.01, ^∗∗∗^*p* < 0.001 vs. SCI group.

To determine whether GSP can exert positive effects on functional recovery, rats were subjected to BBB score and footprint analysis. We found that hindlimb function in rats was lost immediately following SCI and gradually recovered over time (Figure 3(a)). At 28 days postinjury (dpi), the BBB scores of the SCI and GSP-treated groups were 8 and 13, respectively. Starting at 7 dpi, the score of the GSP-treated group was remarkably higher than that of the SCI group. Consistent with this, our footprint analysis results (Figure 3(b)) showed that the hindlimb movement of rats in the GSP-treated group was relatively coordinated, whereas the SCI rats showed obvious hindlimb dragging. These data indicate that GSP can improve locomotor function in SCI rats.

### 3.2. Effect of GSP on Neural Function In Vivo

Histological and morphological changes in rats were evaluated by HE and IF staining. We found that the spinal cords in the SCI group had large cavities that were significantly narrowed by GSP (Figure 3(c)). Next, we stained the cells for NeuN (a neuronal marker) to investigate the effects of GSP on the neurons. As shown in Figure 3(d), more NeuN-positive cells were observed in the GSP-treated group. GFAP was used to assess astrocyte activation. We observed increased GFAP expression in the SCI group, and this increase was markedly attenuated by GSP (Figure 3(e)). These results suggest that GSP can increase neuronal survival and inhibit astrocyte activation following SCI.

### 3.3. Effect of GSP on Microglia Polarisation In Vivo

**Figure 4:**
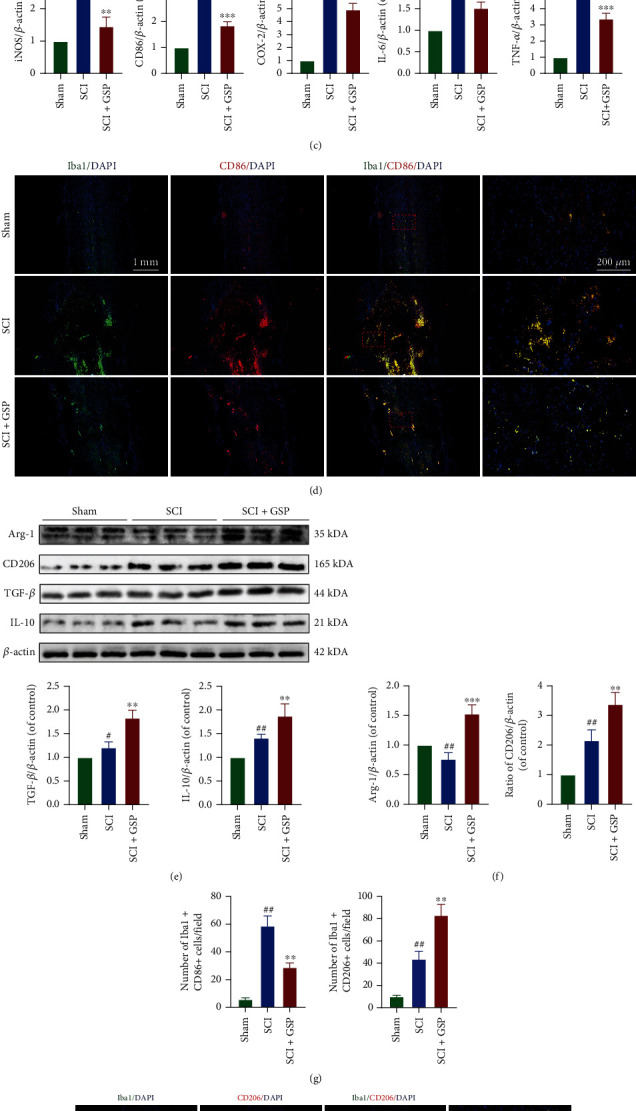
Effect of GSP on microglial polarisation *in vivo*. (a) The M1/M2-related mRNA expressions were measured by qRT-PCR (*n* = 3/group). (b, c, e, f) Representative WB and quantitative data of M1/M2-related protein levels in each group. (*n* = 3/group). (d, g, h) Representative IF and quantitative data of CD86/CD206 at 14 dpi and (*n* = 3/group). ^#^*p* < 0.05, ^##^*p* < 0.01 vs. sham group. ^∗^*p* < 0.05, ^∗∗^*p* < 0.01, or ^∗∗∗^*p* < 0.001 vs. SCI group.

At 14 dpi, qRT-PCR was used to detect the mRNA expression of inflammatory cytokines in the spinal cord tissues. GSP significantly decreased the expression of proinflammatory cytokines (TNF-*α*, iNOS, and COX-2) and elevated the expression of anti-inflammatory cytokines (Arg-1, TGF-*β*, and IL-10) (Figure 4(a)). Next, we investigated whether GSP could regulate microglial polarisation following SCI as microglia have two different phenotypes. The expressions of iNOS, CD86, TNF-*α*, IL-6, COX-2, Arg1, TGF-*β*, CD206, and IL-10 were analyzed by WB (Figures 4(b), 4(c), 4(e), and 4(f)). The GSP-treated group showed lower M1-related protein expression and higher M2-related protein expression than the SCI group. We used CD86, CD206, and Iba1 (a specific marker of microglia) for IF to evaluate microglial polarisation in each group (Figures 4(d) and 4(h)). As shown in Figure 4(g), the GSP-treated group tended to have more CD206-positive and fewer CD86-positive microglia than the SCI group at 14 dpi. These results suggest that GSP has a significant anti-inflammatory effect and can polarise M1 to M2 microglia in rats following SCI.

### 3.4. GSP Suppressed Neuronal Apoptosis In Vivo

**Figure 5:**
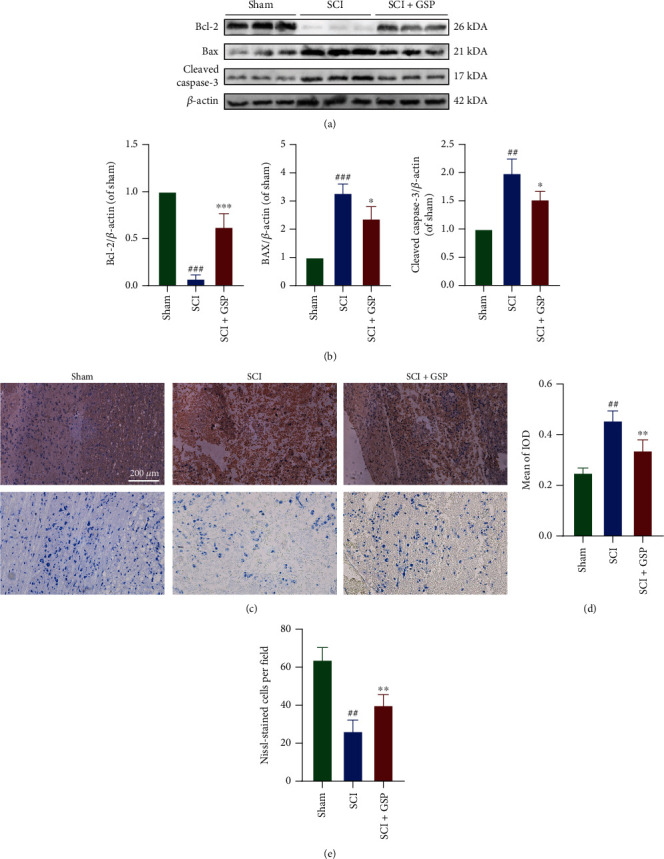
GSP inhibit apoptosis *in vivo*. (a, b) Representative WB and quantitative data of Bax, Bcl-2, and cleaved caspase-3 in the different groups at 14 dpi (*n* = 3 rats in each group). (c, d) Representative IHC staining and Nissl staining in the different groups at 14 dpi. (d) Quantitative data of cleaved caspase-3. (*n* = 3, with 5 images for each rat). (e) Quantitative data of the number of Nissl-stained cells at 14 days after SCI (*n* = 3 rats in each group). ^##^*p* < 0.01 or ^###^*p* < 0.001 vs. sham group. ^∗^*p* < 0.05, ^∗∗^*p* < 0.01, or ^∗∗∗^*p* < 0.001 vs. SCI group.

The relationship between neuronal apoptosis and local inflammation has been previously reported [36]. Sustained microglial activation can cause neurological impairments by inducing neuronal apoptosis [37]. To determine the apoptosis of neurons and whether GSP prevents neuronal apoptosis in rats with SCI, we assayed the levels of apoptosis-related proteins. The WB results (Figures 5(a) and 5(b)) suggested that the expression of antiapoptotic Bcl-2 was downregulated, but proapoptotic Bax and cleaved caspase-3 were upregulated in the SCI versus sham group, all of which could be reversed by GSP treatment. The results of IHC for cleaved caspase-3 (Figures 5(c) and 5(d)) were consistent with those of WB, suggesting that GSP could prevent neuronal apoptosis after SCI in rats.

### 3.5. Effect of GSP on BV2 Cell Viability

**Figure 6:**
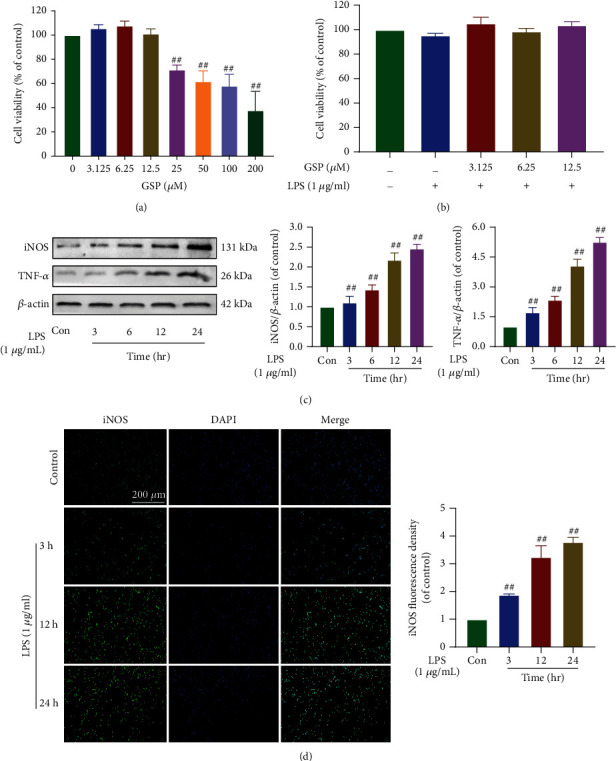
Effect of GSP on the viability and effect of LPS on microglial polarisation. (a) Effects of on cell viability. (b) Effects of GSP and LPS on cell viability. (c) Representative WB and quantitative data of iNOS and TNF-*α* in each group. (d) IF and quantitative data of iNOS. ^##^*p* < 0.01 vs. control group.

To determine whether GSP has a similar therapeutic effect *in vitro* as observed *in vivo*, LPS was added to induce an inflammatory microenvironment and simulate SCI *in vitro.* The CCK-8 assay was used to determine whether GSP affected the viability of BV2 cells. The results (Figure 6(a)) showed that GSP at concentrations lower than 25 *μ*M caused no detectable cytotoxicity in BV2 cells. Therefore, we employed GSP concentrations of 3.125–12.5 *μ*M in subsequent experiments. BV2 cells were sequentially treated with LPS (100 ng/mL, 24 h) and various concentrations of GSP (3.125, 6.25, 12.5, and 24 h). The results (Figure 6(b)) indicated that after being treated with 3.125, 6.25, and 12.5 *μ*M GSP and 100 ng/mL LPS, the viability of BV2 cells showed no significant difference.

### 3.6. Effects of LPS on Microglial Polarisation and Release of Proinflammatory Mediators

Several previous studies have demonstrated that LPS (100 ng/mL) can induce neuroinflammation and polarise microglia to the M1 phenotype [38, 39]. Therefore, in the follow-up experiment, we chose to use LPS at 100 ng/mL. Our results (Figures 6(c) and 6(d)) suggest that the expression levels of TNF-*α* and iNOS increased in a time-dependent manner after LPS stimulation. Moreover, the iNOS expression detected by IF was consistent with that observed using WB (Figure 6(e)). Since 100 ng/mL LPS was sufficient to induce M1 polarisation of microglia, we used 100 ng/mL LPS to treat BV2 cells in subsequent experiments.

### 3.7. Effect of GSP on Microglia Polarisation In Vitro

**Figure 7:**
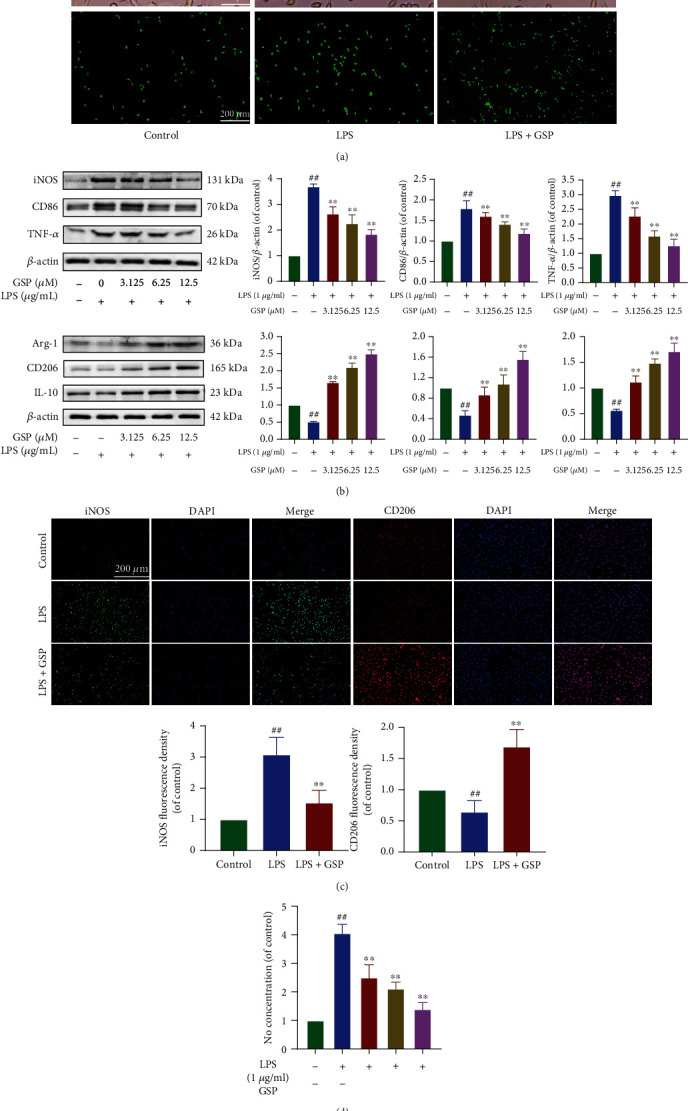
Effect of GSP on microglial polarisation *in vitro*. (a) Morphological results and IF for Iba1 in each group. Scale bar = 200 *μ*m. (b) Representative WB and quantitative data of M1/M2-related proteins. (c) IF and quantitative data of iNOS and CD206 in each group. (d) NO concentration of each group. ^##^*p* < 0.01 vs. control group. ^∗∗^*p* < 0.01 vs. LPS-treated group.

Morphological changes and IF for Iba1 were used to evaluate the effects of GSP (12.5 *μ*M) on LPS-induced microglia. We found that resting BV2 cells had small cell bodies and long processes, showed a spindle shape, and acquired an amoeba-like morphology with short and thick cell bodies after LPS treatment. Nonetheless, these changes could be reversed by GSP (Figure 7(a)). To further investigate whether GSP (12.5 *μ*M) had the same effects *in vitro*, the expression levels of M1/M2-related markers in each group were measured (Figure 7(b)). GSP treatment reduced the expression of M1 microglial markers, similar to the effect of IF on iNOS (Figure 7(c)) and NO production (Figure 7(d)). GSP significantly upregulated the M2 microglial markers (Figure 7(b)). Similarly, the immunofluorescence intensity of CD206 (Figure 7(c)) was lower following LPS treatment but became stronger after GSP treatment. These *in vitro* results indicate that GSP can polarise microglia from the M1 to M2 phenotype, confirming the results observed *in vivo*.

### 3.8. Effect of GSP on M1 Microglia-Induced Neuronal Apoptosis In Vitro

**Figure 8:**
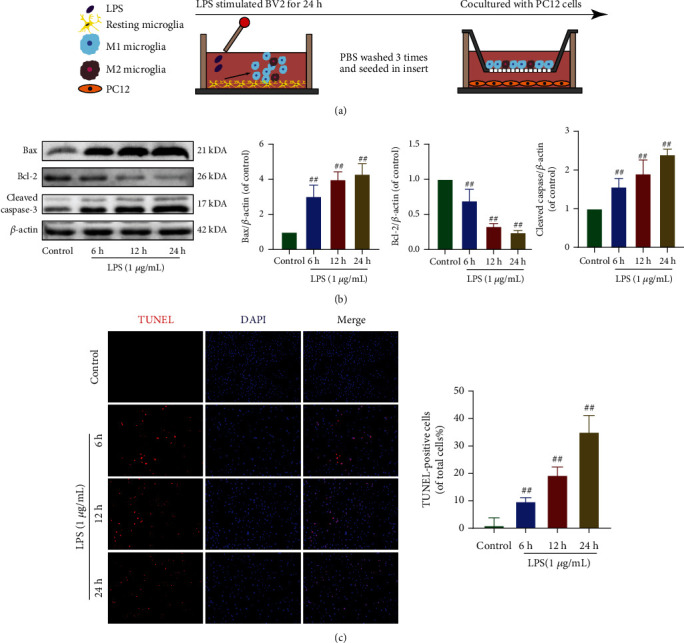
Effect of M1 microglia on neuronal apoptosis. (a) Schematic of cell treatments. (b) Representative WB and quantitative data of apoptosis-related proteins. (c) Apoptosis of PC12 was detected by TUNEL (scale bar: 200 *μ*m). ^##^*p* < 0.01 vs. control group.

To determine the relationship between microglial polarisation and neuronal apoptosis, we cocultured LPS-BV2 and PC12 cells. The cells were then treated as shown in Figure 8(a). The results of TUNEL and WB analyses of apoptosis-related proteins (Figures 8(b) and 8(c)) showed that apoptosis of PC12 cells was induced by M1-BV2 cells.

**Figure 9:**
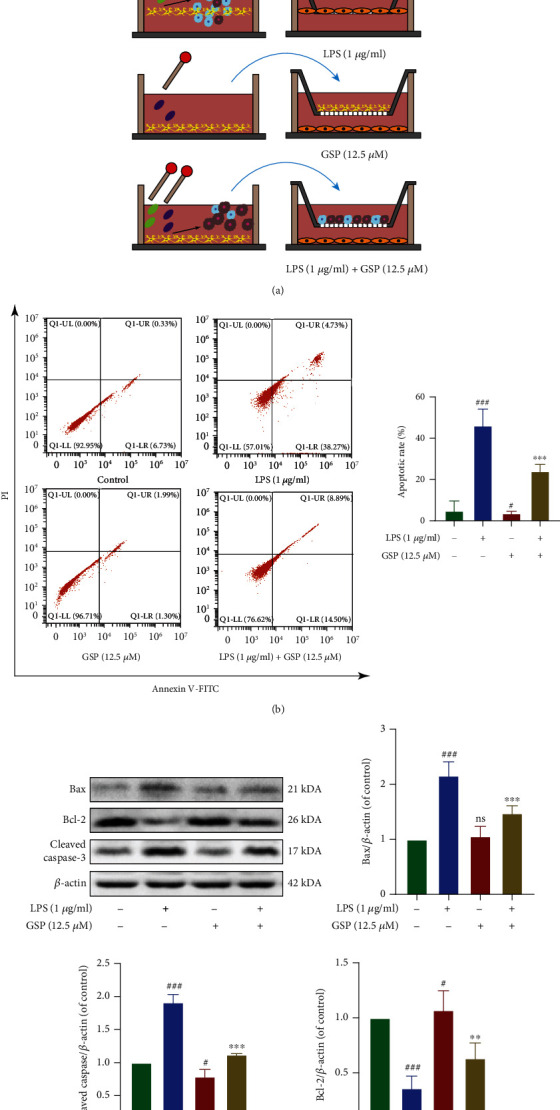
The antiapoptotic effect of GSP. (a) BV-2 cell treatments and the coculture system. (b) Apoptosis rates were measured by flow cytometry. (c) Representative WB and quantitative data of apoptosis-related proteins in each group. ^#^*p* < 0.05 or ^###^*p* < 0.001 vs. control group. ^∗∗^*p* < 0.01 or ^∗∗∗^*p* < 0.001 vs. LPS-treated group.

To evaluate whether GSP affects M1 microglia-induced neuronal apoptosis, we cocultured PC12 cells with differentially treated BV2 cells (Figure 9(a)). The apoptotic rate in monocultured PC12 cells was 7.05%, which decreased to 3.29% when cocultured with GSP-pretreated BV2 cells. When cocultured with LPS-treated BV2 cells, the apoptosis rate of PC12 cells increased significantly to 42.09% and recovered to 23.39% when BV2 cells were pretreated with GSP (Figure 9(b)). Similar trends were observed in WB analysis of apoptosis-related proteins (Figure 9(c)). Taken together, these results indicate that GSP can attenuate apoptosis of neurons induced by M1 microglia.

### 3.9. GSP Regulates Microglial Polarisation by Targeting TLR4-Mediated Signaling

**Figure 10:**
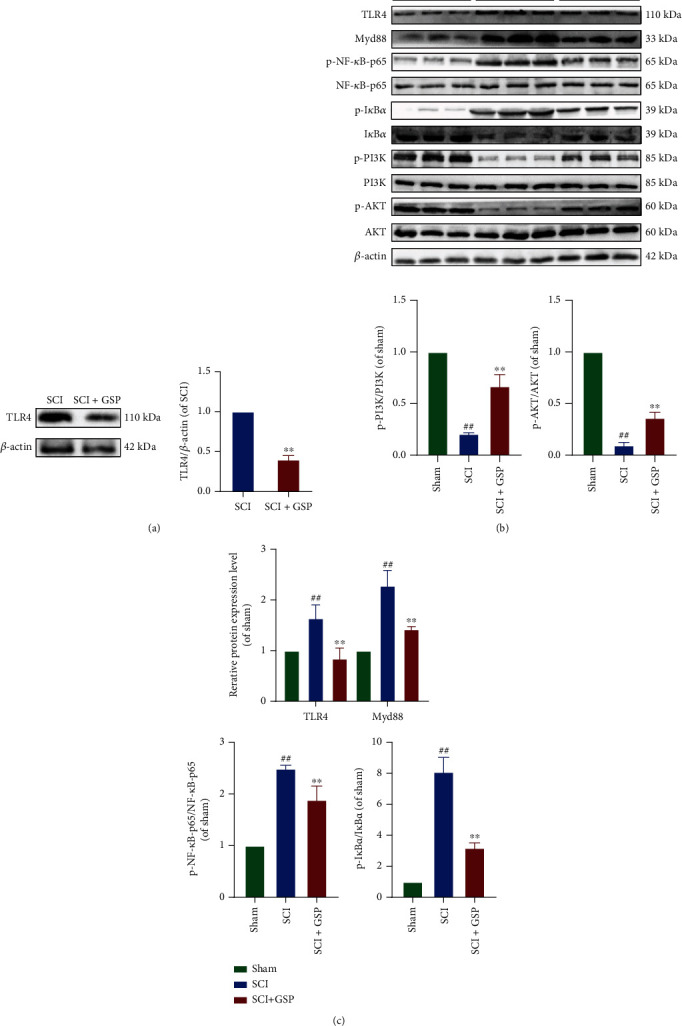
Effect of GSP on TLR4/NF-*κ*B/PI3K/AKT signaling cascades *in vivo*. (a) Representative WB and quantitative analysis of TLR4. (b, c) Representative WB and quantitative analysis of TLR4/MyD88/NF-*κ*B/PI3K/AKT signaling cascades in each group. ^##^*p* < 0.01 vs. sham group. ^∗∗^*p* < 0.01 vs. SCI group.

Both *in vivo* and *in vitro* studies revealed that GSP promotes functional recovery and shifts microglial polarisation from the M1 to M2 phenotype. To elucidate the mechanisms underlying the effects of GSP, we determined the expression of TLR4 after SCI. The WB results suggested that the expression of TLR4 was upregulated in the SCI group but downregulated following GSP treatment. This indicated that GSP could inhibit TLR4. NF-*κ*B and PI3K/AKT are downstream molecules of TLR4. Therefore, we determined the effects of GSP after SCI. In our *in vivo* study, the WB results showed that p-NF-*κ*B-p65 was remarkably inhibited, but p-PI3K and p-AKT (downstream targets of p-PI3K) were activated in the GSP-treated group compared to the SCI group (Figures 10(b) and 10(c)).

**Figure 11:**
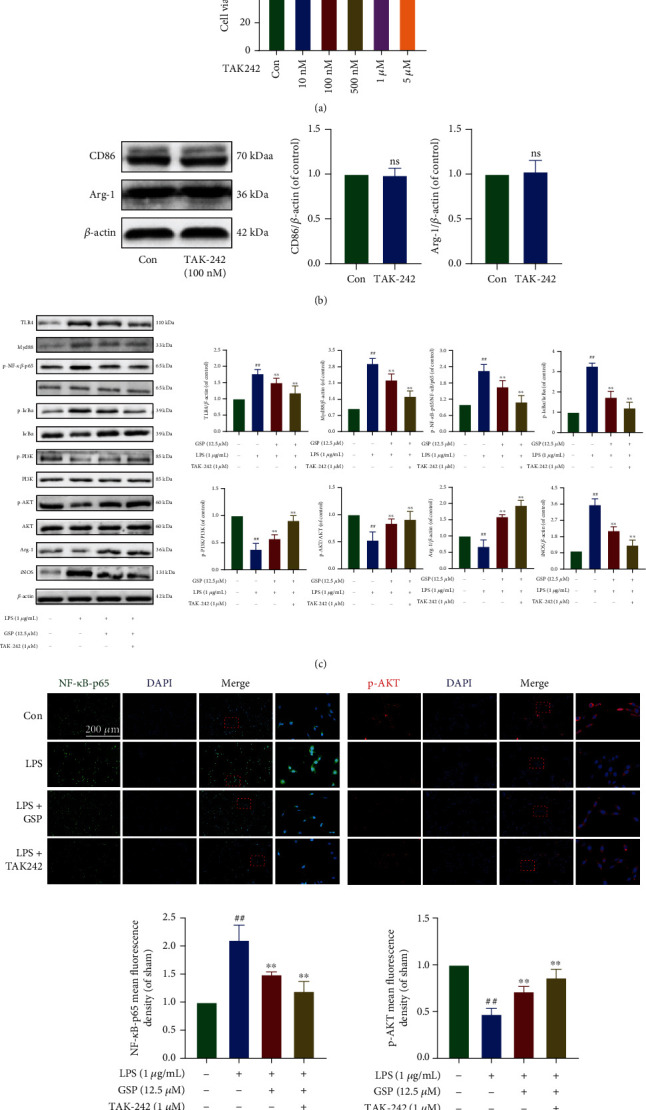
Effect of GSP on TLR4/NF-*κ*B/PI3K/AKT signaling cascades *in vitro*. (a) Effects TAK242 on cell viability. (b) Representative WB and quantitative analysis of CD86 and Arg-1. (c) Representative WB and quantitative data of TLR4/MyD88/NF-*κ*B/PI3K/AKT signaling cascades in each group. (d) IF and quantitative data of p-NF-*κ*B-p65 and p-AKT in each group. ^##^*p* < 0.01 vs. control group. ^∗∗^*p* < 0.01 vs. LPS-treated group.

In our *in vitro* study, TAK242 was used to explore the potential crosstalk between these two pathways after GSP treatment. First, we evaluated whether TAK242 affected BV2 cell viability and found that when the TAK242 concentration was below 500 nM, there was no obvious cytotoxicity (Figure 11(a)). In our preliminary experiment, TAK242 (100 nM) could not polarise BV2 to the M1 or M2 phenotype (Figure 11(b)). Therefore, in the follow-up experiments, we added TAK242 (100 nM) to BV2 cells for 1 h before GSP treatment and found that LPS significantly upregulated the expression of TLR4, MyD88, and p-NF-p65 and downregulated the expression of p-PI3K and p-AKT. However, both GSP (12.5 *μ*M) and TAK242 reversed this trend, and the effect in the inhibitor group was more obvious (Figure 11(c)). Moreover, the IF results were consistent with those obtained using WB (Figure 11(d)). Hence, the above data demonstrates that GSP could promote the polarisation of BV2 cells to the M2 phenotype by targeting the TLR4/Myd88/NF-*κ*B/PI3K/AKT signaling cascades, thus playing a neuroprotective role.

## 4. Discussion

SCI remains a major medical problem worldwide because of its high disability and mortality rates and remains a heavy burden on the patient's family and society. The complexity of the pathological process of SCI has created significant obstacles for the current treatments. Current treatment methods focus on inhibiting neuroinflammation in the secondary injury, thus creating a beneficial microenvironment for neurogenesis and axonal regeneration. Currently, SCI treatment cannot completely restore impaired function. The good news is that most SCI cases involve contusions, traction, or compression injuries rather than physical transection of the spinal cord, and these incomplete SCI can be treated [40].

Proanthocyanidins are pluripotent molecules that can be isolated from many plant species and have been shown to promote health and prevent disease [41–44]. After being metabolized, proanthocyanidins can cross the blood-brain/spinal barrier that prevent most drugs from reaching the CNS [45, 46]. Zhou et al. found that proanthocyanidins could promote functional recovery following SCI by inhibiting ferroptosis [47]. Our previous study demonstrated that proanthocyanidins could inhibit H_2_O_2_-induced apoptosis in PC12 cells [48]. Nonetheless, to date, no study has evaluated the potential effects of proanthocyanidins on microglial polarisation following SCI. Therefore, this study is aimed at exploring whether GSP has a protective effect against SCI and its potential mechanism. We established a rat model of SCI and hypothesized that GSP has a therapeutic effect. First, we evaluated locomotor function recovery in rats. We found that GSP significantly promoted functional recovery. The number of NeuN-positive cells decreased remarkably after SCI; however, this neuronal loss was reduced after GSP treatment. Activated astrocytes can cause glial scarring and affect axonal regeneration. Our IF results indicated that the GFAP expression was upregulated after SCI, and this phenomenon was markedly attenuated by GSP. Taken together, the findings of our preliminary *in vivo* studies demonstrated that GSP could eliminate astrocyte activation and promote functional behavioral recovery in rats following SCI.

As a resident cell type of the CNS, microglia are very important for achieving CNS homeostasis and maintaining normal neuronal function under healthy conditions and are key regulators of SCI and repair. Like macrophages, microglia, as a type of highly plastic cell, can differentiate into M1 and M2 phenotypes. The M1 phenotype expresses various proinflammatory factors (TNF-*α*, NO, and iNOS). The M2 phenotype is characterized by the expression of anti-inflammatory mediators such as IL-10, Arg1, and CD206 [49–51]. Microglia can create a microenvironment conducive to SCI recovery by modulating M1/M2 polarisation. Because microglia can cause neurotoxic or neuroprotective effects by dynamically switching between M1 and M2 phenotypes following stimulation, inhibiting overactivated M1 polarisation seems to be a feasible strategy for neuroprotection. However, there is evidence that inhibition of the M1 phenotype alone is unlikely to provide overall benefits [52, 53]. Compared to simply inhibiting M1 polarisation, the timely conversion of M1 to M2 microglia is considered a more promising strategy for treating SCI [54]. In our *in vivo* study, we used M1/M2-related markers to characterize microglial polarisation. We found that the M1 marker expression decreased, whereas the M2 marker expression increased, indicating that GSP could regulate microglial polarisation after SCI.

In the secondary injury of SCI, in addition to neurons, apoptosis also occurs in other cells in the CNS that leads to further loss of neurological function, thus creating obstacles to repair. Inhibition of neuronal apoptosis can promote functional and pathological recovery and prevent permanent neurological impairments [55]. The BCL-2 family of proteins is essential apoptosis regulatory proteins, including proapoptotic (e.g., Bax and cleaved caspase-3) and antiapoptotic (e.g., Bcl-2) proteins. Higher Bax/Bcl-2 ratios can form ion channels that activate cleaved caspase-3, which induces apoptosis [56]. Our *in vivo* results showed that GSP inhibited neuronal apoptosis.

To determine whether GSP has a similar therapeutic effect *in vitro* as observed *in vivo*, we used BV2 cells to simulate primary microglia for follow-up experiments. The rationale of BV-2 as a substitute for primary microglia has been verified previously [57]. Activated microglia can induce apoptosis in surrounding neurons [58–60]. Since we found that GSP inhibited neuronal apoptosis *in vivo*, we wondered whether it was mediated, at least partially, by inhibiting the activation of M1 microglia. Therefore, we evaluated the effects of M1 microglia on neuronal apoptosis *in vitro* using a coculture system and found that GSP alleviated neuronal apoptosis by inhibiting M1 polarisation in microglia.

LPS, a component of the outer membrane of gram-negative bacteria, is a classical TLR4 agonist that polarises microglia to the proinflammatory M1 phenotype and reduces M2 polarisation, thus aggravating inflammation [16, 61]. LPS-induced microglial models have been widely adopted in studies on microglial polarisation [62, 63]. Therefore, we stimulated BV2 cells with LPS to simulate neuroinflammation *in vitro* and found that LPS caused NO release and increased the expression of TNF-*α*, CD86, and iNOS in BV2 cells. Nevertheless, GSP reversed this trend and significantly upregulated M2-related markers, which suggested that GSP could polarise microglia from the M1 to the M2 phenotype.

TLR4 is mainly expressed in microglia of the CNS [64, 65]. TLR4-dependent microglial activation is crucial in SCI [66, 67]. The biological effects mediated by TLR4 are mainly related to its downstream signaling pathway, and the activation of TLR4 is essential for microglial M1 polarisation [67]. In our *in vivo* study, the expression of TLR4 decreased significantly after GSP treatment. This indicated that GSP may inhibit the TLR4-mediated signaling pathway. Taken together, these results suggest that the effects of GSP on microglia-induced neuroinflammation or neuroapoptosis may be related to the TLR4-mediated signaling pathway.

**Figure 12:**
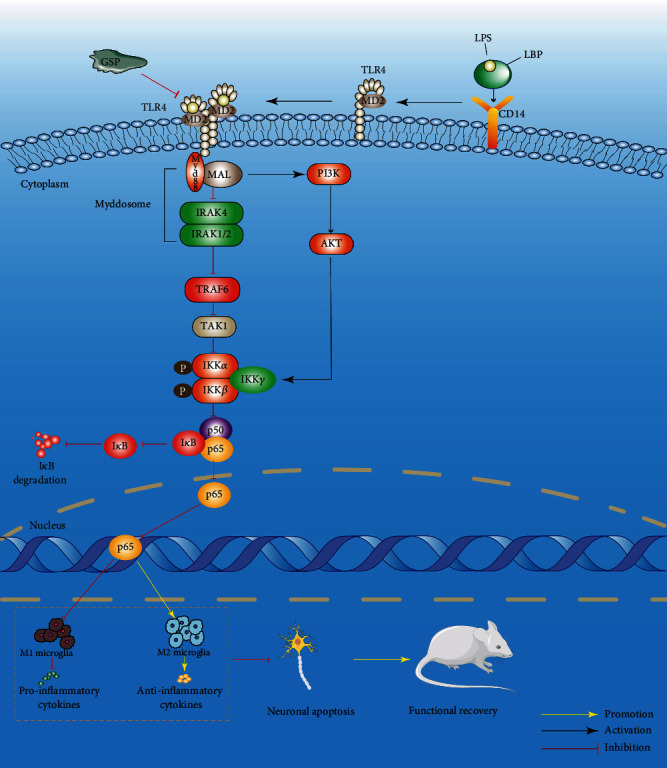
Schematic diagram of GSP regulate microglial polarisation through TLR4/NF-*κ*B/PI3K/AKT signaling pathway.

After binding to TLR4, LPS induces downstream signaling pathways of TLR4, such as NF-*κ*B [68]. NF-*κ*B normally binds to I*κ*B and remains in the cytoplasm in its inactive state. Upon activation, the rapid degradation of I*κ*B leads to its dissociation from the NF-*κ*B dimer (p65/p50). The dissociation of I*κ*B from the NF-*κ*B dimer can expose the DNA binding signal on the p65 subunit and the translocation signal on the p50 subunit, which results in p65/p50 dimer translocation from the cytoplasm to the nucleus [69, 70]. The PI3K/AKT pathway is another major regulator of neuroinflammation. When activated, it contributes to functional recovery following SCI [71] and promotes microglial polarisation towards the M2 phenotype [72]. There is growing evidence of crosstalk between the TLR4/Myd88/NF-*κ*B and PI3K/AKT signaling pathways [73, 74]. Moreover, activation of PI3K/AKT signaling can inhibit the TLR4/NF-*κ*B signaling pathway (Figure 12) [75]. Our results strongly indicate that GSP can regulate microglia towards the M2 phenotype, which may involve the two signaling pathways.

The results *in vivo* suggested that the NF-*κ*B pathway was strongly activated following SCI, whereas PI3K/AKT pathway activity was inhibited. This trend was reversed by GSP treatment. To further explore the effects of GSP on these two pathways, we detected changes in related protein levels *in vitro*. Compared to the LPS group, the NF-*κ*B pathway was inhibited, and the PI3K/AKT pathway was activated after GSP treatment. One strategy for inhibiting LPS-mediated signaling pathways is to block their receptors. Therefore, we blocked TLR4 with TAK242. We found that both TAK242 and GSP effectively reduced the high activity of NF-*κ*B induced by LPS and upregulated PI3K/AKT signaling-related protein expression. Compared to TAK242, the regulation of GSP is limited or milder but sufficient to reduce LPS-induced neuroinflammation. We demonstrated that GSP regulates microglial polarisation by inhibiting the TLR4-mediated NF-*κ*B and PI3K/AKT signaling pathways. Nevertheless, further experiments should be performed to explain the precise underlying mechanism of crosstalk between TLR4/NF*κ*B and PI3K/AKT signaling pathways.

In summary, GSP can regulate microglial polarisation, thereby reducing neuronal apoptosis and improving functional recovery after SCI by inhibiting TLR4. Therefore, the TLR4/MyD88/NF-*κ*B/PI3K/AKT signaling cascade is a suitable target for treating SCI. However, this study did not explore the long-term side effects of GSP, and the exact mechanism needs to be further explored.

## 5. Conclusion

GSP inhibited neuroinflammation and neuronal apoptosis by regulating microglial polarisation. Thus, GSP plays a neuroprotective role, which may be achieved by inhibiting the NF-*κ*B signaling pathway and activating the PI3K/AKT signaling pathway mediated by TLR4. This study demonstrated the potential of GSP as a therapeutic SCI drug.

## Figures and Tables

**Table 1:** Primer sequences for qRT-PCR.

Gene	Forward primer sequence	Reverse primer sequence
COX-2	TCATAAGCGAGGACCTGG	GGTGGCATACATCATCAGAC
iNOS	CACCGAGATTGGAGTTCG	GGAGCACAGCCACATTG
TNF-*α*	TGGAACTGGCAGAAGAGG	GAACTGATGAGAGGGAGGC
Arg-1	TGGCAGAGGTCCAGAAGTG	GGAGTGTTGATGTCAGTGTGAGC
IL-10	TGCTATGCTGCCTGCTC	TGGCTGAACCAAGGAGACG
TGF-*β*	TGGCTGAACCAAGGAGAC	CTCTGTGGAGCGTTGATTTCC
GAPDH	TGTGTCCGTCGTGGATCTGA	TGGCTGTTGAAGTAGCAGGAG

## Data Availability

The data generated in this study can be obtained from the corresponding author upon request.
